# Supplementary material for: Unveiling the Bioactive Potential of the Invasive Jellyfish Phyllorhiza punctata Through Integrative Transcriptomic and Proteomic Analyses
Source: Biomolecules. 2025 Aug 4;15(8):1121. doi: 10.3390/biom15081121 (PMC12383608; doi:10.3390/biom15081121)
Supplement: Supplementary file 1 [file biomolecules-15-01121-s001.zip › Suplementary Material/FileS3/PEPFOLD4/XP_065069680_9513/clusters.html]

 PEP-FOLD clusters


Download archive of all models  here   

|  |  |  |
| --- | --- | --- |
| # | file | sOPEP |
| # cluster 1 - Size: 2 |  |  |
|  | XP\_065069680\_9513-model1 | -6.6478 |
|  | XP\_065069680\_9513-model1.1 | -6.6478 |
| # cluster 2 - Size: 27 |  |  |
|  | XP\_065069680\_9513-model2 | -6.6478 |
|  | XP\_065069680\_9513-model2.1 | -6.6478 |
|  | XP\_065069680\_9513-model2.2 | -6.6478 |
|  | XP\_065069680\_9513-model2.3 | -6.6478 |
|  | XP\_065069680\_9513-model2.4 | -6.6478 |
|  | XP\_065069680\_9513-model2.5 | -6.6478 |
|  | XP\_065069680\_9513-model2.6 | -6.6478 |
|  | XP\_065069680\_9513-model2.7 | -6.6478 |
|  | XP\_065069680\_9513-model2.8 | -6.6478 |
|  | XP\_065069680\_9513-model2.9 | -6.6478 |
|  | XP\_065069680\_9513-model2.10 | -6.6478 |
|  | XP\_065069680\_9513-model2.11 | -6.6478 |
|  | XP\_065069680\_9513-model2.12 | -6.6478 |
|  | XP\_065069680\_9513-model2.13 | -6.6478 |
|  | XP\_065069680\_9513-model2.14 | -6.6478 |
|  | XP\_065069680\_9513-model2.15 | -6.6478 |
|  | XP\_065069680\_9513-model2.16 | -6.6478 |
|  | XP\_065069680\_9513-model2.17 | -6.6478 |
|  | XP\_065069680\_9513-model2.18 | -6.6478 |
|  | XP\_065069680\_9513-model2.19 | -6.6478 |
|  | XP\_065069680\_9513-model2.20 | -6.6478 |
|  | XP\_065069680\_9513-model2.21 | -6.6478 |
|  | XP\_065069680\_9513-model2.22 | -6.6478 |
|  | XP\_065069680\_9513-model2.23 | -6.6478 |
|  | XP\_065069680\_9513-model2.24 | -6.6478 |
|  | XP\_065069680\_9513-model2.25 | -6.6478 |
|  | XP\_065069680\_9513-model2.26 | -6.6478 |
| # cluster 3 - Size: 7 |  |  |
|  | XP\_065069680\_9513-model3 | -6.6478 |
|  | XP\_065069680\_9513-model3.1 | -6.6478 |
|  | XP\_065069680\_9513-model3.2 | -6.6478 |
|  | XP\_065069680\_9513-model3.3 | -6.6478 |
|  | XP\_065069680\_9513-model3.4 | -6.6478 |
|  | XP\_065069680\_9513-model3.5 | -6.6478 |
|  | XP\_065069680\_9513-model3.6 | -6.6478 |
| # cluster 4 - Size: 2 |  |  |
|  | XP\_065069680\_9513-model4 | -6.6478 |
|  | XP\_065069680\_9513-model4.1 | -6.6478 |
| # cluster 5 - Size: 6 |  |  |
|  | XP\_065069680\_9513-model5 | -6.6478 |
|  | XP\_065069680\_9513-model5.1 | -6.6478 |
|  | XP\_065069680\_9513-model5.2 | -6.6478 |
|  | XP\_065069680\_9513-model5.3 | -6.6478 |
|  | XP\_065069680\_9513-model5.4 | -6.6478 |
|  | XP\_065069680\_9513-model5.5 | -6.6478 |
| # cluster 6 - Size: 11 |  |  |
|  | XP\_065069680\_9513-model6 | -6.6478 |
|  | XP\_065069680\_9513-model6.1 | -6.6478 |
|  | XP\_065069680\_9513-model6.2 | -6.6478 |
|  | XP\_065069680\_9513-model6.3 | -6.6478 |
|  | XP\_065069680\_9513-model6.4 | -6.6478 |
|  | XP\_065069680\_9513-model6.5 | -6.6478 |
|  | XP\_065069680\_9513-model6.6 | -6.6478 |
|  | XP\_065069680\_9513-model6.7 | -6.6478 |
|  | XP\_065069680\_9513-model6.8 | -6.6478 |
|  | XP\_065069680\_9513-model6.9 | -6.6478 |
|  | XP\_065069680\_9513-model6.10 | -6.6478 |
| # cluster 7 - Size: 6 |  |  |
|  | XP\_065069680\_9513-model7 | -6.6478 |
|  | XP\_065069680\_9513-model7.1 | -6.6478 |
|  | XP\_065069680\_9513-model7.2 | -6.6478 |
|  | XP\_065069680\_9513-model7.3 | -6.6478 |
|  | XP\_065069680\_9513-model7.4 | -6.6478 |
|  | XP\_065069680\_9513-model7.5 | -6.6478 |
| # cluster 8 - Size: 9 |  |  |
|  | XP\_065069680\_9513-model8 | -6.6478 |
|  | XP\_065069680\_9513-model8.1 | -6.6478 |
|  | XP\_065069680\_9513-model8.2 | -6.6478 |
|  | XP\_065069680\_9513-model8.3 | -6.6478 |
|  | XP\_065069680\_9513-model8.4 | -6.6478 |
|  | XP\_065069680\_9513-model8.5 | -6.6478 |
|  | XP\_065069680\_9513-model8.6 | -6.6478 |
|  | XP\_065069680\_9513-model8.7 | -6.6478 |
|  | XP\_065069680\_9513-model8.8 | -6.6478 |
| # cluster 9 - Size: 7 |  |  |
|  | XP\_065069680\_9513-model9 | -6.6478 |
|  | XP\_065069680\_9513-model9.1 | -6.6478 |
|  | XP\_065069680\_9513-model9.2 | -6.6478 |
|  | XP\_065069680\_9513-model9.3 | -6.6478 |
|  | XP\_065069680\_9513-model9.4 | -6.6478 |
|  | XP\_065069680\_9513-model9.5 | -6.6478 |
|  | XP\_065069680\_9513-model9.6 | -6.6478 |
| # cluster 10 - Size: 5 |  |  |
|  | XP\_065069680\_9513-model10 | -6.6478 |
|  | XP\_065069680\_9513-model10.1 | -6.6478 |
|  | XP\_065069680\_9513-model10.2 | -6.6478 |
|  | XP\_065069680\_9513-model10.3 | -6.6478 |
|  | XP\_065069680\_9513-model10.4 | -6.6478 |
| # cluster 11 - Size: 5 |  |  |
|  | XP\_065069680\_9513-model11 | -6.6478 |
|  | XP\_065069680\_9513-model11.1 | -6.6478 |
|  | XP\_065069680\_9513-model11.2 | -6.6478 |
|  | XP\_065069680\_9513-model11.3 | -6.6478 |
|  | XP\_065069680\_9513-model11.4 | -6.6478 |
| # cluster 12 - Size: 8 |  |  |
|  | XP\_065069680\_9513-model12 | -6.6478 |
|  | XP\_065069680\_9513-model12.1 | -6.6478 |
|  | XP\_065069680\_9513-model12.2 | -6.6478 |
|  | XP\_065069680\_9513-model12.3 | -6.6478 |
|  | XP\_065069680\_9513-model12.4 | -6.6478 |
|  | XP\_065069680\_9513-model12.5 | -6.6478 |
|  | XP\_065069680\_9513-model12.6 | -6.6478 |
|  | XP\_065069680\_9513-model12.7 | -6.6478 |
| # cluster 13 - Size: 3 |  |  |
|  | XP\_065069680\_9513-model13 | -6.6478 |
|  | XP\_065069680\_9513-model13.1 | -6.6478 |
|  | XP\_065069680\_9513-model13.2 | -6.6478 |
| # cluster 14 - Size: 2 |  |  |
|  | XP\_065069680\_9513-model14 | -6.6478 |
|  | XP\_065069680\_9513-model14.1 | -6.6478 |
|  |  |  |
